# The Multitasking *Fasciola gigantica* Cathepsin B Interferes With Various Functions of Goat Peripheral Blood Mononuclear Cells *in vitro*

**DOI:** 10.3389/fimmu.2019.01707

**Published:** 2019-07-23

**Authors:** Dan Chen, Ai-Ling Tian, Jun-Ling Hou, Jie-Xi Li, XiaoWei Tian, Xiao-Dan Yuan, Xiangrui Li, Hany M. Elsheikha, Xing-Quan Zhu

**Affiliations:** ^1^State Key Laboratory of Veterinary Etiological Biology, Key Laboratory of Veterinary Parasitology of Gansu Province, Lanzhou Veterinary Research Institute, Chinese Academy of Agricultural Sciences, Lanzhou, China; ^2^College of Veterinary Medicine, Northwest A&F University, Yangling, China; ^3^College of Veterinary Medicine, Nanjing Agricultural University, Nanjing, China; ^4^Faculty of Medicine and Health Sciences, School of Veterinary Medicine and Science, University of Nottingham, Loughborough, United Kingdom

**Keywords:** *Fasciola gigantica*, cysteine protease, cathepsin B, immunomodulation, host-parasite interaction

## Abstract

Cathepsin B, a lysosomal cysteine protease, is thought to be involved in the pathogenesis of *Fasciola gigantica* infection, but its exact role remains unclear. In the present study, a recombinant *F. gigantica* cathepsin B (rFgCatB) protein was expressed in the methylotrophic yeast *Pichia pastoris*. Western blot analysis confirmed the reactivity of the purified rFgCatB protein to serum from *F. gigantica*-infected goats. The effects of serial concentrations (10, 20, 40, 80, and 160 μg/ml) of rFgCatB on various functions of goat peripheral blood mononuclear cells (PBMCs) were examined. We demonstrated that rFgCatB protein can specifically bind to the surface of PBMCs. In addition, rFgCatB increased the expression of cytokines (IL-2, IL-4, IL-10, IL-17, TGF-β, and IFN-γ), and increased nitric oxide production and cell apoptosis, but reduced cell viability. These data show that rFgCatB can influence cellular and immunological functions of goat PBMCs. Further characterization of the posttranslational modification and assessment of rFgCatB in immunogenicity studies is warranted.

## Introduction

Fasciolosis, caused by the liver flukes *Fasciola gigantica* (*F. gigantica*) and *Fasciola hepatica* (*F. hepatica*), is an important parasitic disease with a worldwide distribution (1). These liver flukes can infect a wide range of mammalian species, including livestock, wild animals, and humans. *F. gigantica* is responsible for significant economic losses in the buffalo-producing countries due to its chronic morbidity and adverse effects on the animal health, fecundity, and productivity (2). Adding to the challenge is the emerging evidence of *F. gigantica* resistance against albendazole and rafoxanide (3), and the lack of a commercial vaccine. Better understanding of the mechanisms and factors that shape the immuno-pathogenesis of fasciolosis may ultimately facilitate the design of new immunotherapeutic strategies for efficient treatment of fasciolosis.

*Fasciola* spp. employ multiple strategies to evade the host immune response using various molecules in their excretory/secretory (E/S) products (4–7), such as cathepsin B and L proteases (8–10). At least six types of cathepsin B have been detected in the immature and invasive stages of *Fasciola*, and have been shown to play roles in the pathogenesis of fasciolosis (9, 11, 12). Due to their immune-modulatory functions, cysteine proteases such as cathepsin B have attracted significant attention as potential immuno-therapeutic targets to control liver fluke infection (9).

In *F. hepatica*, cathepsins interact with host immune cells and skew the immune response toward a non-protective Th2-mediated/regulatory response (13). In *F. gigantica*, cathepsin B2 and B3 digest host substrates, such as immunoglobulin, fibronectin, and collagen (14–16). Also, cathepsin B5, expressed in immature and adult stages of *F. gigantica*, can digest host proteins (17). The recombinant proteins (rFgCatB2 and rFgCatB3) can elicit a mixed Th1/Th2 immune response with the predominance of Th2 cytokines (16). Despite significant efforts, information about the modulatory effects of *F. gigantica* cathepsin B on the host innate immune cells is still limited.

In the present study, the gene encoding *F. gigantica* cathepsin B was cloned and expressed in *Pichia pastoris*. We characterized the modulatory effects of the purified recombinant *F. gigantica* cathepsin B protein (rFgCatB) on various functions of goat peripheral blood mononuclear cells (PBMCs), including cytokine secretion, cell viability, nitric oxide (NO) production, and apoptosis. We show that rFgCatB induces a mixed Th1/Th2/Th17 immune response and significantly influences other functions of goat PBMCs. Our findings demonstrate the feasibility of including rFgCatB protein in a vaccination trial against fasciolosis.

## Materials and Methods

### Ethics Statement

All experimental protocols were reviewed and approved by the Animal Administration and Ethics Committee of Lanzhou Veterinary Research Institute, Chinese Academy of Agricultural Sciences (Permit No. 2018-012). All animal experiments were performed in strict compliance with the Animal Ethics Procedures and Guidelines of the People's Republic of China. All efforts were made to minimize the suffering of animals, and daily health checks were performed during the entire experiments.

### Animals and Collection of Blood Cells

Eight local crossbred goats (4- to 7-month-old) were obtained from Laboratory Animal Center of Lanzhou Veterinary Research Institute, Chinese Academy of Agriculture Science. All goats were kept in-door and dewormed with albendazole and ivermectin tablets (Xining Fengyuan Agricultural and Animal Sci-Tech Company, Xining, China) to eliminate any potential existing helminth infection. Before and 2 weeks after treatment, fecal samples from each goat were microscopically examined for helminth eggs. This analysis showed that all goats used in the study are free from any prior or current helminth infection. For the production of antisera, four female New Zealand rabbits (3-month-old) were purchased from Laboratory Animal Center of Lanzhou Veterinary Research Institute, Chinese Academy of Agriculture Science and were housed under specific-pathogen-free conditions, with access to food and water *ad libitum*. Peripheral venous blood samples were collected from three healthy goats and peripheral blood mononuclear cells (PBMCs) and monocytes were isolated and cultured as previously described (7).

### Parasite Preparation

Adult flukes were harvested from the gall bladder of naturally infected buffaloes at local slaughterhouses in Guangxi Zhuang Autonomous Region, PR China. The harvested flukes were washed with phosphate buffered saline (PBS, pH7.4) and immediately used for RNA isolation or stored at −80°C with RNA stabilizer for future use. The flukes were identified as *F. gigantica* based on amplification and sequencing of the internal transcribed spacer 2 (ITS-2) of the ribosomal DNA (18). Sequence alignment showed no difference between the ITS-2 sequence obtained in our study and the ITS-2 sequence obtained previously from *F. gigantica* samples collected from buffaloes in Guangxi province (GenBankaccession No. AJ557569).

### Cloning and Characterization of *FgCatB* Gene

Due to the lack of genomics data on *F. gigantica*, we have searched *F. hepatica* E/S product's dataset produced by liquid chromatography-tandem mass spectrometry (LC-MS/MS), *F. hepatica* cDNA library available from previous proteomic studies, and the BLASTx search protein database (https://blast.ncbi.nlm.nih.gov/Blast.cgi), in order to identify homologous cathepsin B protein sequences. This analysis identified *F. hepatica* cathepsin B protein isoform (FhCatB) sequence (GenBank accession No. Z22768.1), which was used to design primers to amplify *F. gigantica* cathepsin B (*FgCatB*) gene sequence. Total *F*. *gigantica* RNA was isolated from 30 mg of adult *F*. *gigantica* flukes using Trizol reagent (Invitrogen, San Diego, USA). The first-strand cDNA was synthesized by reverse transcription polymerase chain reaction (RT-PCR) using RevertAid First Strand cDNA Synthesis Kit (Thermo Scientific, (EU) Lithuania). The cDNA was used as a template to amplify *FgCatB* gene using two oligonucleotide primers: 5′- CCG GAA TTC CAT ATG AGC TTA CTG ATC TCC AGC-3′ (forward) and 5′- ATT TGC GGC CGC CTC GAG TTG GGG TAA TTT TGG C-3′ (reverse). The oligonucleotide primers were synthesized with the *EcoRI* (forward) or *Not* I (reverse) restriction site underlined. The resulting amplified *FgCatB* gene product was digested with *Eco*R I and *Not* I, and cloned into pMD19-T (Takara, Dalian, Liaoning, China). The recombinant plasmid was transformed into *Trans5*α chemically competent cells (TransGen Biotech, Beijing, China). Several positive clones were selected and sequenced by GenScript (Nanjing, Jiangsu, China) to confirm the correct insertion/orientation of *FgCatB* gene in the vector. The signal peptide, transmembrane helices (TMHs) and *N*-glycosylation sites of the *FgCatB* sequence were predicted using SignalP 5.0 Server (http://www.cbs.dtu.dk/services/SignalP/), TMHMM Server v. 2.0 (http://www.cbs.dtu.dk/services/TMHMM/), and NetNGlyc 1.0 Server (http://www.cbs.dtu.dk/services/NetNGlyc/), respectively.

### Expression of rFgCatB Protein

A single positive clone containing the *FgCatB* gene was selected and the *FgCatB* gene fragment was sub-cloned into pPIC9K vector. A carboxyl-terminal His6 tag and appropriate restriction sites were included in the expression plasmid to enable purification. The plasmid designated as pPIC9K-*FgCatB* was linearized with *Sal* I and electroporated into the methylotrophic yeast *P*. *pastoris* GS115 strain using a GenePulser X cell TM (Bio-Rad, Hercules, California, USA). Positive recombinant *P. pastoris* clones containing the insert were selected for expression by inoculating into 15 ml of buffered complex medium containing glycerol (BMGY). The inoculated BMGY medium (1% [wt/vol] yeast extract, 2% [wt/vol] peptone, 1% [wt/vol] yeast nitrogen base, 1% [wt/vol] glycerol, 0.00004% [wt/vol] biotin, and 0.1 M potassium phosphate [pH 6.0]) in 100 ml conical flasks was incubated at 28°C with vigorous shaking for 24 h. The cells were harvested by centrifugation (250 × g for 10 min), resuspended in 20 ml of buffered complex medium containing methanol (BMMY; BMGY medium with 1% methanol substituted for glycerol). The culture was allowed to continue growing for 4 days. During *FgCatB* gene expression induction period, methanol was added every 24 h to maintain a final concentration of 1% (v/v). The cells were pelleted by centrifugation (2,500 × g at 4°C for 10 min) and the culture supernatant was harvested for protein extraction.

### Purification of Recombinant *F. gigantica* Cathepsin B (rFgCatB) Protein

The yeast culture supernatant containing rFgCatB protein was concentrated by centrifugation at 4,000 × g for 15 min using Amicon^®^ Ultra 10 K centrifugal filter device. The concentrated supernatant was purified using the His GaviTrap Kit (GE Healthcare, Buckinghamshire, UK) at 4°C. The rFgCatB protein was eluted with elution buffer (20 mM PBS, 0.5 M NaCl, 500 mM imidazole, PH 7.4) and dialyzed against 1 × PBS to remove imidazole. The concentration of the protein was determined by the Bradford method, using bovine serum albumin (BSA) as the standard. Purified proteins were stored at −80°C until further analysis.

### Preparation of Antibodies

Four, 4- to 7-month-old, goats were challenged orally with 250 viable encysted metacercariae of *F. gigantica*. After 3 months, the goat sera containing anti-*F. gigantica* antibodies were collected. Serum was collected from one healthy naïve goat (negative control) and stored frozen at −80°C. Specific antibodies against rFgCatB protein were produced by immunizing three New Zealand rabbits with rFgCatB. For primary immunization, 200 μg of the purified rFgCatB protein mixed with complete Freund's adjuvant (1:1) were injected subcutaneously into multiple sites at the back of the rabbits, followed by four booster doses with 100 μg of the recombinant protein in incomplete Freund's adjuvant at 2-week intervals. One week after the last injection, antisera against rFgCatB was collected. In the meantime, serum was collected from one healthy rabbit (negative control) and stored frozen at −80°C.

### SDS-PAGE and Western Blotting

The isolated protein (20 μg) was separated on 12% sodium dodecyl sulfate (SDS)-polyacrylamide gel electrophoresis (PAGE) gels and stained with Coomassie Blue. The protein migrated on gels as a “blurred” smear without showing the expected band size, indicating that rFgCatB is a glycoslyated protein. Therefore, rFgCatB was deglycosylated under denaturing conditions using Protein Deglycosylation Mix II (New England Biolab^®^ Inc., USA), as per the manufacturer's instructions. The deglycosylated rFgCatB protein was resolved on 12% SDS-PAGE gels, followed by Coomassie Blue staining. Also, the deglycosylated rFgCatB was transferred onto Hybond-C extra nitrocellulose membrane (Amersham, London, UK). The membrane was blocked using 5% skim milk in Tris-buffered saline containing 0.1% Tween-20 (TBST) for 2 h at ambient temperature, followed by incubation with primary antibodies (antiserum from goats experimentally infected with *F*. *gigantica*) for 12 h at 4°C (1:100 in TBST). After being washed three times (5 min each) with TBST, the membrane was incubated with HRP-conjugated rabbit anti-goat IgG (Sigma, St. Louis, MO, USA) for 1 h at 37°C (1:2500 in TBST). Finally, freshly prepared 3,3′-diaminobenzidine (DAB, Sigma) was used as a chromogenic substrate to visualize the immunoreaction.

### Measurement of rFgCatB Activity

The enzyme activity of rFgCatB was measured using Cathepsin B Activity Assay Kit (Abcam, ab65300) according to the manufacturer's instructions. Briefly, 50 μg of rFgCatB protein was adjusted to 50 μL per well with cell lysis buffer for experimental samples in a 96-well plate. Fifty microliters of blank cell lysis buffer were used for measuring background. Next, 50 μL CB Reaction Buffer followed by 2 μL of cathepsin B substrate Ac-RR-AFC (amino-4-trifluoromethyl coumarin) were added to each well. The plates were incubated at 37°C for 2 h protected from light, and fluorescence from the cathepsin B-cleaved substrate was measured at excitation/emission (Ex/Em) = 400/505 nm using a fluorescent microplate reader (Thermo scientific, Varioskan LUX Multimode Microplate Reader). The relative enzyme activity of rFgCatB was represented as the fold increase in the fluorescence intensity compared with the cathepsin B inhibitor-treated control.

### Immunofluorescence Detection of rFgCatB Protein Binding to Goat PBMCs

Goat PBMCs were incubated with 10 μg/ml of rFgCatB in a humidified atmosphere of 5% CO_2_ at 37°C for 1 h. The rFgCatB-treated cells were fixed with 4% paraformaldehyde at ambient temperature for 15 min, washed three times in PBS (5 min each), and subsequently treated with blocking solution (4% BSA in PBS) for 1 h to minimize background staining. rFgCatB-treated or non-treated control PBMCs were incubated with rabbit anti-rFgCatB antibody (dilution, 1:100) for 12 h at 4°C and washed three times in PBS (5 min each). Cells were stained with Cy3 conjugated goat anti-rabbit IgG secondary antibody (dilution, 1:500) (Beyotime, Haimen, Jiangsu, China) for 1 h at 37°C. Hoechst 33342 (Invitrogen, Eugene, Oregon, USA) was used to stain the nucleus. Localization of rFgCatB was visualized using a Zeiss laser scanning confocal microscope (LSM710, Zeiss, Jena, Germany) at 100 × magnification and images were analyzed using Zen 2012 imaging software.

### Cytokine Analysis

The concentrations of cytokines were evaluated in the supernatant of 5 ×10^4^ PBMCs seeded into 24-well tissue culture plates in 1 ml RPMI 1640 medium/well. Serial concentrations (10, 20, 40, 80, and 160 μg/ml) of rFgCatB protein or equal volume of PBS (control) were added to the wells. The culture plates were incubated at 37°C with 5% CO_2_ for 72 h. The supernatants were collected and the concentrations of interleukin-2 (IL-2), IL-4, IL-10, IL-17, interferon gamma (IFN-γ), and transforming growth factor-beta (TGF-β) were determined using goat enzyme linked immunosorbent assay (ELISA) kits (Mlbio, Shanghai, China) as per the manufacturer's instructions.

### The Effect of rFgCatB Protein on Cell Viability

The effect of rFgCatB protein on the viability of PBMCs was examined by a CCK-8 assay (Beyotime, Haimen, Jiangsu, China). This assay is based on the measurement of the reduction of a water-soluble tetrazolium salt WST-8 by dehydrogenases in viable cells. Briefly, PBMCs (10^4^ cells/100 μl RPMI 1640 medium/well) seeded into 96-well tissue culture plates were incubated with serial concentrations (10, 20, 40, 80, or 160 μg/ml) of rFgCatB protein or equal volume of PBS (control) at 37°C in a humidified atmosphere with 5% CO_2_. Following 48 h incubation, 10 μl of CCK-8 reagent were added per well and the culture plates were further incubated under the same conditions for 4 h in protected from light. The optical density at 450 nm (OD_450_) was measured using a microplate reader (Bio-Rad, Hercules, California, USA). The OD_450_ of control wells (cells incubated with PBS) was set as 100% and the cell viability index was calculated using the formula: OD_450_ rFgCatB /OD_450_ control.

### Determination of Nitric Oxide (NO)

PBMCs were seeded into a 24-well tissue culture plate at 5 ×10^4^ cells/well in 1 ml RPMI 1640 medium. Cells were incubated with various concentrations (10, 20, 40, or 80 μg/ml) of rFgCatB protein or equal volume of PBS (control) at 37°C with 5% CO_2_ for 24 h. The NO level in PBMC culture supernatant was determined by measuring the concentrations of nitrite using the Total Nitric Oxide Assay Kit (Beyotime, Haimen, Jiangsu, China). A microplate reader (Bio-Rad, Hercules, California, USA) was used to measure the absorbance values at 540 nm (OD_540_). NO levels were calculated using a standard curve generated by 0 to 80 μM/L sodium nitrites.

### Evaluation of the Apoptotic Effect of rFgCatB Protein

Flow cytometry analysis (BD Biosciences, San Jose, California, USA) was carried out to evaluate the apoptosis in PBMCs using the Annexin V-FITC kit (Beyotime, Haimen, Jiangsu, China). PBMCs seeded into a 24-well tissue culture plate at 5 ×10^4^ cells/well in 1 ml RPMI 1640 medium were incubated with the above mentioned concentrations of rFgCatB protein or equal volume of PBS (control) at 37°C with 5% CO_2_ for 24 h. Then, cells were washed twice with PBS, re-suspended in binding buffer, and stained with Annexin V and Propidium Iodide (PI) according to the manufacturer's instructions. The results were analyzed using FlowJo 10.

### Statistical Analysis

The statistical analyses were performed by one-way ANOVA, followed by a Dunnett's test or *t*-test using GraphPad Premier 6.0 software package (GraphPad Prism, San Diego, California, USA). Significant differences between rFgCatB-treated and control groups are indicated in the figures by asterisks (^*^, *P* < 0.05; ^**^, *P* < 0.01; ^***^, *P* < 0.001 or ^****^, *P* < 0.0001). Data were presented as means ± standard deviation (SD). All experiments were repeated at least three separate times.

## Results

### Identification, Cloning, and Expression of rFgCatB Protein

We performed multiple searches to identify homologous of *F. gigantica* cathepsin B sequence in the genome of *F. hepatica*. This analysis identified *F. hepatica* cathepsin B-like protease (GenBank accession no. Z22768.1) sequence, which was used to design 5′ and 3′ primers to amplify the *FgCatB* gene. The cDNA fragment of *FgCatB* was successfully cloned into the pMD19-T cloning vector and the positive pMD19-T-*FgCatB* clones were subjected to nucleic acid sequencing. The obtained *FgCatB* sequence has been submitted to GenBank under accession number MN038412. The amino acid sequence similarity search showed that cathepsin B endopeptidase of *F. hepatica* (THD22097.1) has the highest similarity (100% homology) to *FgCatB*. The ORF contained 1,038 base-pair (bp) and encoded 345 amino acids. The deduced amino acid sequence predicts the existence of a signal peptide, two *N*-linked glycosylation sites and four protein kinase C phosphorylation sites, however, no TMH was detected.

### SDS-PAGE and Western Blotting Analysis

**Figure 1:**
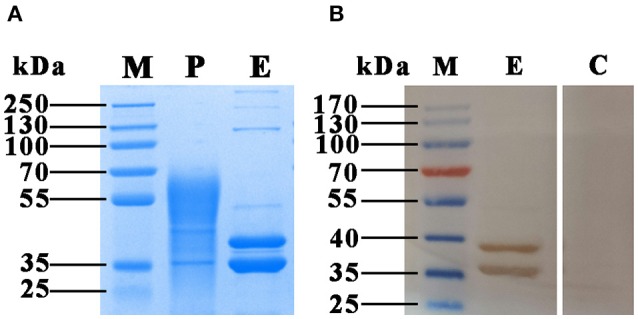
SDS-PAGE and Western blotting analysis of the rFgCatB protein purified from the culture supernatant of *P. pastoris*. **(A)** Proteins were resolved on 12% acrylamide gels and stained with Coomassie brilliant blue R250. Lane M, protein molecular weight marker; Lane P, purified rFgCatB appeared heterogeneous and ranged in size from ~36 to 70 kDa; Lane E, rFgCatB treatment with endoglycosidase H (Endo H) revealed two distinct bands at ~38 and 36 kDa. **(B)** The protein treatment with Endo H was run under non-reducing conditions, and visualized by immunodetection using specific antibodies and enhanced chemiluminescence. Lane M, protein molecular weight marker; Lane E, was loaded with rFgCatB digested with Endo H. Serum from *F. gigantica*-infected goats detected ~ 38 and 36 kDa bands; Lane C, was loaded with rFgCatB, which did not react with serum of healthy goats.

To verify the presence of FgCatB protein in *F. gigantica*-derived material, *FgCatB* gene fragment was cloned into the pPIC9K vector and the positive clones, designated as pPIC9K-*FgCatB*, were transformed into *P. pastoris*. The recombinant protein (rFgCatB) was successfully isolated from the culture supernatant of *P. pastoris*. The expected molecular mass of rFgCatB is 38.2 kDa, however after 72 h of induction with 1% methanol the purified protein exhibited a heterogeneous molecular mass ranging from ~36–70 kDa on SDS-PAGE. Two bands of approximately 38 and 36 kDa appeared after deglycosylation using endoglycosidase H (Endo H) treatment, which cleaves high-mannose *N*-linked glycans (Figure 1A). Western blot analysis using serum from *F. gigantica*-infected goats confirmed the specificity of the two bands, which were absent when the Western blot was probed with serum from healthy goats (Figure 1B).

### Enzymatic Activity of rFgCatB

**Figure 2:**
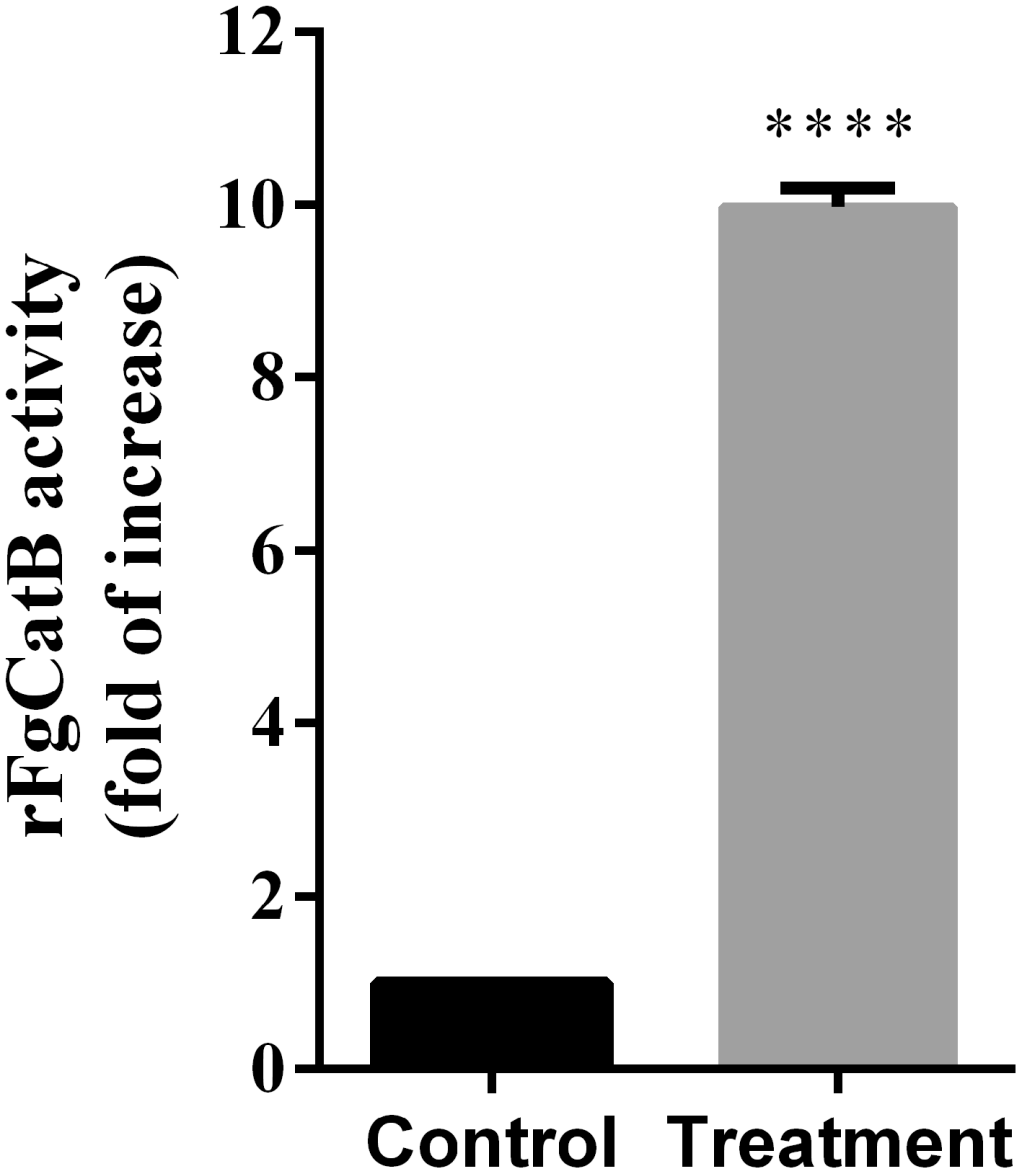
*Fasciola gigantica*-derived rFgCatB enzymatic activity was determined by examining its ability to cleave the fluorescent synthetic substrate RR-AFC to release free AFC. The enzyme activity of rFgCatB was measured by Cathepsin B Activity Assay Kit as described in the materials and methods and the result showed a high activity of the rFgCatB (*t*-test, *t* = 72.68, *P* < 0.0001). Asterisks indicate statistical significance between rFgCatB sample and cathepsin B inhibitor-treated control sample (^****^P < 0.0001).

The activity of cathepsin B was examined using the Fluorometric ab65300 assay kit. *Fasciola gigantica*-derived rFgCatB enzymatic activity was determined by measuring its ability to cleave the fluorescent synthetic substrate RR-AFC to release free AFC. The results showed that the enzyme activity of rFgCatB is several fold higher than that of the control, confirming the functional activity for cathepsin B (Figure 2).

### Binding Affinity of rFgCatB Protein to Goat PBMCs

**Figure 3:**
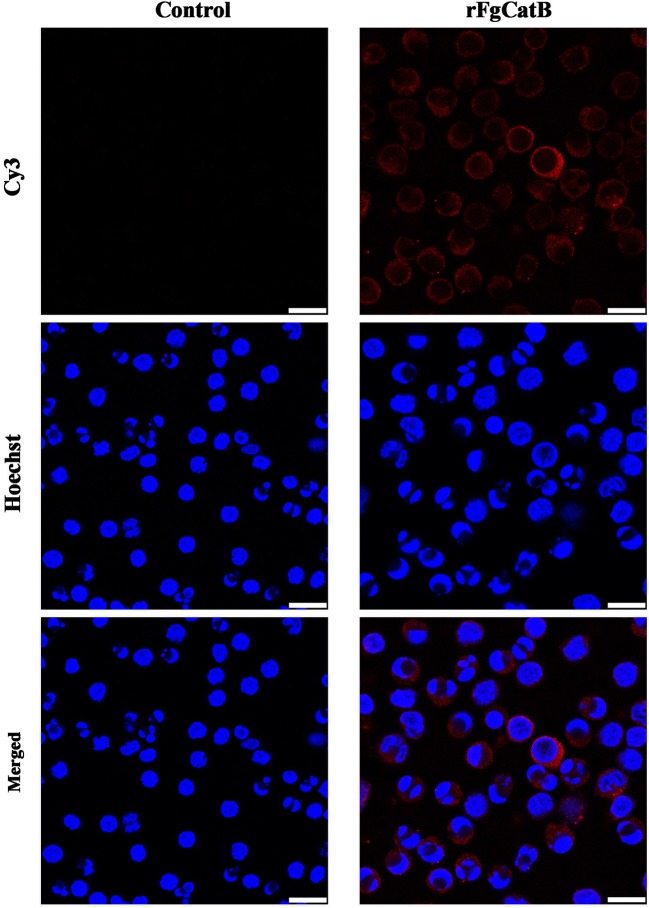
Localization of *Fasciola gigantica*-derived rFgCatB protein on the surface of PBMCs. Cells were treated with rFgCatB and incubated with rabbit anti-rFgCatB primary antibody. Hoechst (blue) and Cy3-conjugated secondary antibody (red) were used to stain host cell nuclei and rFgCatB protein, respectively. Surface localization was observed in rFgCatB-treated cells, whereas no staining was detected in untreated (control) cells. Scale bars, 10 μm.

Indirect immunofluorescence staining was used to determine the binding affinity of rFgCatB protein to the surface of PBMCs. By incubating rFgCatB-treated PBMCs with rabbit anti-rFgCatB antibodies, the localization of the red Cy3 conjugated goat anti-rabbit IgG secondary antibody on the cell surface was observed, suggesting successful binding of rFgCatB to the surface of PBMCs (Figure 3). There was no fluorescence observed in the untreated control cells.

### rFgCatB Protein Increased Cytokine Production

**Figure 4:**
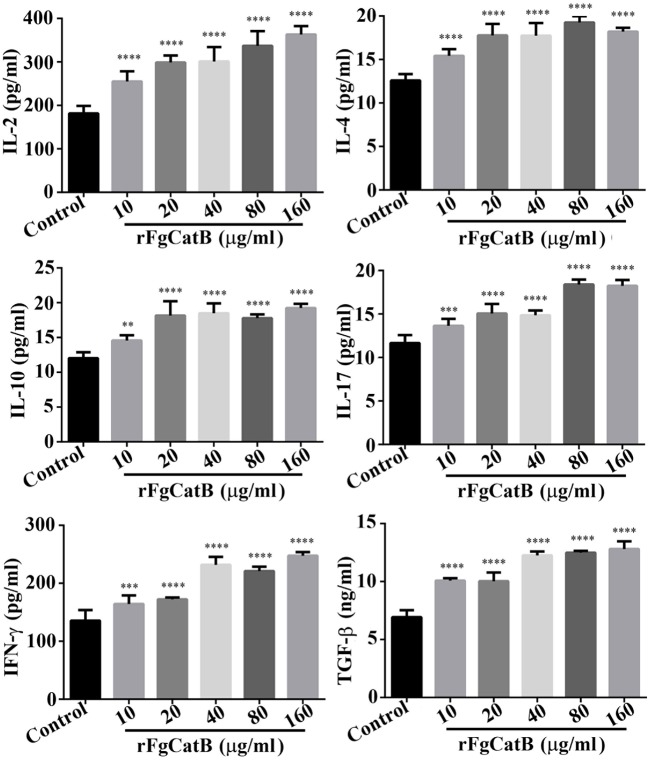
rFgCatB protein stimulated the production of cytokines. Goat PBMCs were incubated for 72 h in the presence of PBS or serial concentrations of rFgCatB protein. The levels of cytokines in the supernatant of cultured PBMCs were quantified by ELISA. Results showed that rFgCatB induced the expression of all examined cytokines in a dose-dependent manner. Graphs represent means ± standard deviations of data from three independent biological replicates (IL-2 (10 μg/ml: *F*_(5, 36)_ = 46.98, *P* < 0.0001; 20 μg/ml: *F*_(5, 36)_ = 46.98, *P* < 0.0001; 40 μg/ml: *F*_(5, 36)_ = 46.98, *P* < 0.0001; 80 μg/ml: *F*_(5, 36)_ = 46.98, *P* < 0.0001; 160 μg/ml: *F*_(5, 36)_ = 46.98, *P* < 0.0001), IL-4 (10 μg/ml: *F*_(5, 36)_ = 43.65, *P* < 0.0001; 20 μg/ml: *F*_(5, 36)_ = 43.65, *P* < 0.0001; 40 μg/ml: *F*_(5, 36)_ = 43.65, *P* < 0.0001; 80 μg/ml: *F*_(5, 36)_ = 43.65, *P* < 0.0001; 160 μg/ml: *F*_(5, 36)_ = 43.65, *P* < 0.0001), IL-10 (10 μg/ml: *F*_(5, 36)_ = 40.51, *P* = 0.0012; 20 μg/ml: *F*_(5, 36)_ = 40.51, *P* < 0.0001; 40 μg/ml: *F*_(5, 36)_ = 40.51, *P* < 0.0001; 80 μg/ml: *F*_(5, 36)_ = 40.51, *P* < 0.0001; 160 μg/ml: *F*_(5, 36)_ = 40.51, *P* < 0.0001), IL-17 (10 μg/ml: *F*_(5, 36)_ = 77.39, *P* = 0.0002; 20 μg/ml: *F*_(5, 36)_ = 77.39, *P* < 0.0001; 40 μg/ml: *F*_(5, 36)_ = 77.39, *P* < 0.0001; 80 μg/ml: *F*_(5, 36)_ = 77.39, *P* < 0.0001; 160 μg/ml: *F*_(5, 36)_ = 77.39, *P* < 0.0001), TGF-β (10 μg/ml: *F*_(5, 36)_ = 137.5, *P* < 0.0001; 20 μg/ml: *F*_(5, 36)_ = 137.5, *P* < 0.0001; 40 μg/ml: *F*_(5, 36)_ = 137.5, *P* < 0.0001; 80 μg/ml: *F*_(5, 36)_ = 137.5, *P* < 0.0001; 160 μg/ml: *F*_(5, 36)_ = 137.5, *P* < 0.0001), and IFN-γ (10 μg/ml: *F*_(5, 36)_ = 96.82, *P* = 0.0003; 20 μg/ml: *F*_(5, 36)_ = 96.82, *P* < 0.0001; 40 μg/ml: *F*_(5, 36)_ = 96.82, *P* < 0.0001; 80 μg/ml: *F*_(5, 36)_ = 96.82, *P* < 0.0001; 160 μg/ml: *F*_(5, 36)_ = 96.82, *P* < 0.0001). Asterisks indicate statistical significance between treated and untreated control PBMCs (^**^P < 0.01; ^***^P < 0.001; ^****^P < 0.0001 compared with control).

To understand how rFgCatB modulates cytokine production of PBMCs, the levels of six cytokines, IL-2, IL-4, IL-10, IL-17, IFN-γ, and TGF-β, were determined. As shown in Figure 4, when PBMCs were treated with serial concentrations of rFgCatB protein, the production of all six cytokines was significantly increased compared with control (PBS-treated) PBMCs.

### Cytotoxic Effect of rFgCatB Protein

**Figure 5:**
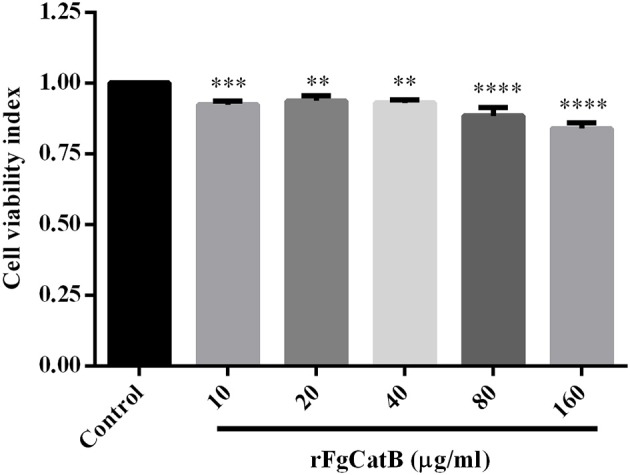
Effect of rFgCatB protein on PBMC viability. Goat PBMCs were treated with PBS or with serial concentrations of rFgCatB protein and incubated for 48 h at 37°C at 5% CO_2_. Viability of cells was determined using CCK-8 assay. Results indicate that rFgCatB protein significantly reduced the viability of PBMCs in a dose-dependent manner. Graphs represent means ± standard deviations of data from three independent biological replicates (10 μg/ml: ANOVA, *F*_(5, 12)_ = 28.50, *P* = 0.0007; 20 μg/ml: ANOVA, *F*_(5, 12)_ = 28.50, *P* = 0.0035; 40 μg/ml: ANOVA, *F*_(5, 12)_ = 28.50, *P* = 0.0015; 80 μg/ml: ANOVA, *F*_(5, 12)_ = 28.50, *P* < 0.0001; 160 μg/ml: ANOVA, *F*_(5, 12)_ = 28.50, *P* < 0.0001). Asterisks indicate significant differences between rFgCatB-treated and PBS-treated control cells (^**^P < 0.01; ^***^P < 0.001; ^****^P < 0.0001 compared with control).

We examined whether rFgCatB protein affects the viability of PBMCs. The CCK-8 assay showed that the viability of PBMCs was remarkably decreased following treatment with rFgCatB protein, at all tested protein concentrations (Figure 5).

### Nitric Oxide (NO) Production

**Figure 6:**
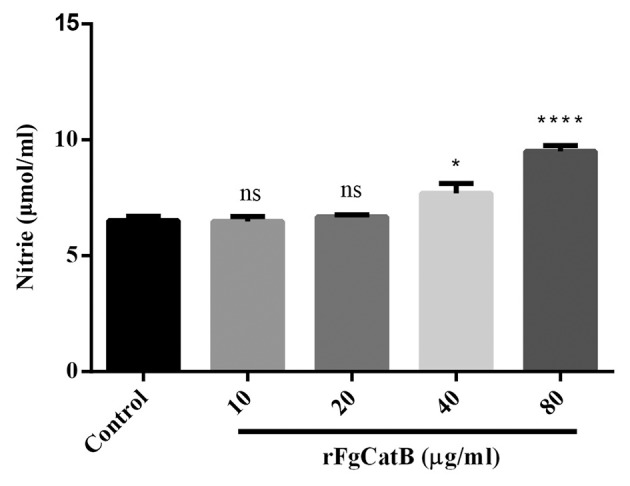
Effects of rFgCatB protein on the production of NO. PBMCs were treated with PBS or with serial concentrations of rFgCatB protein and maintained at 37°C for 24 h. NO concentration was measured by Griess assay. Graphs represent means ± standard deviations of data from three independent biological replicates (10 μg/ml: ANOVA, *F*_(4, 10)_ = 27.91, *P* = 0.9998; 20 μg/ml: ANOVA, *F*_(4, 10)_ = 27.91, *P* = 0.9702; 40 μg/ml: ANOVA, *F*_(4, 10)_ = 27.91, *P* = 0.0220; 80 μg/ml: ANOVA, *F*_(4, 10)_ = 27.91, *P* < 0.0001). Asterisks indicate significant differences between rFgCatB-treated and PBS-treated control cells (^*^p < 0.05; ^****^P < 0.0001; ns, non-significant compared with control).

As shown in Figure 6, compared to the control (PBS-treated PBMCs), NO release was slightly increased in rFgCatB-treated PBMCs at 40 μg/ml and was significantly increased in rFgCatB-treated PBMCs at 80 μg/ml, but not at 10 or 20 μg/ml.

### rFgCatB Protein Induced Cell Apoptosis

**Figure 7:**
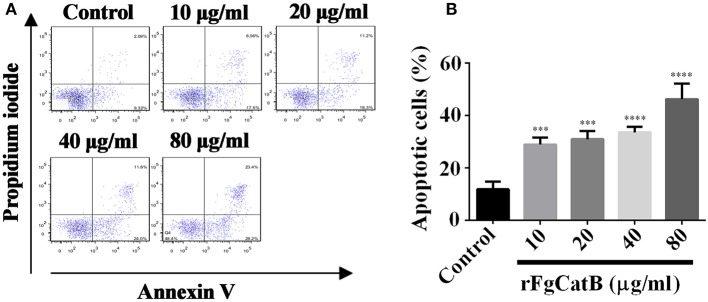
rFgCatB protein induced apoptosis in goat PBMCs. Annexin V/PI staining was used to quantify apoptotic cells by flow cytometry. **(A)** The FACS plot showing apoptosis of PBMCs in response to exposure to rFgCatB protein. **(B)** Apoptotic cells (Annexin V+/PI-) were plotted and compared with the percentage of cell population. Graphs represent means ± standard deviations of data from three independent biological replicates (10 μg/ml: ANOVA, *F*_(4, 10)_ = 34.92, *P* = 0.0006; 20 μg/ml: ANOVA, *F*_(4, 10)_ = 34.92, *P* = 0.0003; 40 μg/ml: ANOVA, *F*_(4, 10)_ = 34.92, *P* < 0.0001; 80 μg/ml: ANOVA, *F*_(4, 10)_ = 34.92, *P* < 0.0001). The asterisks indicate significant differences between rFgCatB-treated and PBS-treated control goat PBMCs (^***^P < 0.001; ^****^P < 0.0001 compared with control).

To explore whether rFgCatB protein induces apoptosis in goat PBMCs, Annexin V-FITC apoptosis assay was used. The rFgCatB protein significantly induced apoptosis in PBMCs at all tested concentrations compared to PBS-treated, control PBMCs (Figure 7). The apoptosis was induced in a dose-dependent manner with the percentage of apoptotic cells treated with rFgCatB at 10, 20, 40, and 80 μg/ml were 28.88 ± 2.631%, 30.95 ± 3.128%, 33.50 ± 2.152%, and 46.17 ± 5.955%, respectively.

## Discussion

In this study, we cloned and expressed the gene encoding cathepsin B of *F. gigantica* in the methylotrophic yeast *P. pastoris*. Although the expected size of the purified rFgCatB protein with six-histidine tag is 38.2 kDa, a significant increase in its molecular weight was observed. Yeast expression system has been known to introduce post-translational modifications such as glycosylation which may affect protein folding. Also, recombinant proteins produced in *P. pastoris* tend to be hypermannosylated. On SDS-PAGE gels, rFgCatB appeared as a group of bands with molecular weights between ~36 and 70 kDa. The observed heterogeneity in the size of the protein may be attributed to *N*-linked glycosylation (19). Deglycosylation of rFgCatB using Endo H revealed ~38 kDa band, which corresponds to the theoretical molecular mass of rFgCatB, and another band with molecular masse of 36 kDa, suggesting the presence of two different glycosylated species (i.e., diglycosylated forms) of rFgCatB, particularly, as both reacted in Western blot. This type of finding has been also reported for cathepsin B from the Asiatic liver fluke *Opisthorchis viverrini* (20).

Our results showed that rFgCatB induced expression of Th1 type cytokines (IL-2 and IFN-γ), Th2 type cytokines (IL-4, IL-10, and TGF-β), and Th17 type cytokine (IL-17), suggesting that rFgCatB can induce a mixed T helper 1 (Th1)-, Th2-, and Th17-type immune response. The high levels of pro-inflammatory cytokines (IL-2 and IFN-γ) and activation of monocytes have been associated with intestinal pathology and release of NO to limit the fluke growth (21–23). On the other hand, high expression of Th2 anti-inflammatory cytokines can facilitate parasite persistence, while minimizing host tissue damage (24–27). For example, the anti-inflammatory cytokine IL-4 inhibits NO production (28) and promotes Th2 differentiation (29), thereby facilitating the production of other anti-inflammatory cytokines (e.g., IL-10 and TGF-β) and inhibiting pro-inflammatory cytokines (e.g., IL-2 and IFN-γ) (30, 31). Also, IL-10 decreases the production of IFN-γ and IL-2 (22, 31).

TGF-β, together with other inflammatory cytokines, can promote Th17 differentiation (32–34). Th17 cells play an important role in host protection against various parasitic infections by recruiting macrophages and neutrophils to infected tissues, and through the modulation of Th1/Th2 balance (34–36). The role of IL-17 in the inflammatory process during *F. gigantica* infection has been reported (37, 38). Interestingly, TGF-β can inhibit T cell proliferation by suppressing the production of IL-2, and inhibiting the differentiation of Th1 and Th2 cells (39).

We have previously shown that *F. gigantica* proteins, rFg14-3-34 and rFgRab10, inhibit cell proliferation, and induce apoptosis and NO production in goat PBMCs (6, 7). The results of the present study lend further support to these previous findings, where rFgCatB was found to bind to the surface of PBMCs similar to what we have demonstrated for rFg14-3-4 and rFgRab10 proteins (6, 7), and to reduce the viability and increase apoptosis of PBMCs. The biological relevance of the pro-apoptotic effect of rFgCatB on PBMCs remains to be determined. However, induction of apoptosis, rather than necrosis, may favor the parasite's persistence because apoptotic cell death does not provoke inflammatory response (40), which can be detrimental to the parasite's survival inside the host.

The antiproliferative and pro-apoptotic effects of E/S products of *Fasciola* spp. on immune cells are some of the strategies used by these liver flukes to hamper immune defenses, leaving the host more vulnerable to infection. *F. hepatica*-derived E/S products have been shown to inhibit the proliferation of sheep lymphoid cells, especially CD4^+^ T lymphocytes (41–43), reduce the proliferation of rat spleen mononuclear cells (44) and induce apoptosis of murine eosinophils and peritoneal macrophages (45, 46). Also, immunosuppression of CD4^+^ T lymphocytes has been observed in *F. hepatica*-infected goats (47). Additionally, *F. hepatica* can induce apoptosis in sheep PBMCs by up-regulating the expression of TNF-α and TNFR1/TNFR2 (48). The induction of apoptosis in sheep eosinophils (49) and peritoneal leucocytes (50) has been suggested to play a role in the pathogenesis of *F. hepatica* by supporting the survival of the juvenile parasites during the migratory and biliary stages of infection.

In summary, our data show that rFgCatB interacts with serum from goats infected with *F*. *gigantica* and accumulates at the surface of PBMCs. The importance of our data resides in the fact that rFgCatB represents a new mechanism for *F*. *gigantica* to evade the host's immune response through modulation of the immune response and biological functions of PBMCs. Exposure of these cells to rFgCatB caused increased production of cytokines (IL-2, IL-4, IL-10, IL-17, TGF-β, and IFN-γ), increased NO production, increased apoptosis, and inhibition of cell viability. Our data provide a proof of concept that rFgCatB is involved *F. gigantica*-interaction with immune cells. In the light of these findings and given that rFgCatB and other *F. gigantica-*derived proteins (e.g., rFg14-3-34 and rFgRab10) can modulate key cellular and immunological functions of goat PBMCs, future work should focus on identifying the appropriate synergistic combinations of these proteins to develop a cocktail vaccine for testing against *F. gigantica* infection.

## Data Availability

All datasets generated for this study are included in the manuscript.

## Ethics Statement

All experimental protocols were reviewed and approved by the Animal Administration and Ethics Committee of Lanzhou Veterinary Research Institute, Chinese Academy of Agricultural Sciences (Permit No. 2018-012). All animal experiments were performed in strict compliance with the Animal Ethics Procedures and Guidelines of the People's Republic of China. All efforts were made to minimize the suffering of animals, and daily health checks were performed during the entire experiments.

## Author Contributions

X-QZ, XL, and HE conceived the idea, planned the experiments, and provided critical feedback. DC performed the experiments, analyzed the data, and drafted the manuscript with the help of HE. A-LT, J-LH, J-XL, XT, and X-DY participated in the implementation of the study. All authors read and approved the final manuscript.

### Conflict of Interest Statement

The authors declare that the research was conducted in the absence of any commercial or financial relationships that could be construed as a potential conflict of interest.
